# Analysis on the Morphology and Interface of the Phosphate Coating Prepared on X39Cr13 and S355J2 Steels

**DOI:** 10.3390/ma17122805

**Published:** 2024-06-08

**Authors:** Monika Gwoździk, Mirosław Bramowicz, Sławomir Kulesza

**Affiliations:** 1 Faculty of Production Engineering and Materials Technology, Czestochowa University of Technology, Armii Krajowej Street 19, 42-201 Czestochowa, Poland; 2 Faculty of Technical Sciences, University of Warmia and Mazury in Olsztyn, Oczapowskiego 11, 10-719 Olsztyn, Poland; mbramowicz@moskit.uwm.edu.pl (M.B.); slawomir.kulesza@uwm.edu.pl (S.K.)

**Keywords:** SEM, image processing, watershed, shape factor, Feret’s diameter

## Abstract

The article presents the results of the characterization of the geometric structure of the surface of unalloyed structural steel and alloyed (martensitic) steel subjected to chemical processing. Prior to phosphating, the samples were heat-treated. Both the surfaces and the cross-sections of the samples were investigated. Detailed studies were made using scanning electron microscopy (SEM), XRD, metallographic microscopy, chemical composition analysis and fractal analysis. The characteristics of the surface geometry involved such parameters as circularity, roundness, solidity, Feret’s diameter, watershed diameter, fractal dimensions and corner frequencies, which were calculated by numerical processing of SEM images.

## 1. Introduction

Phosphating is the most often used surface treatment and finishing process for ferrous and non-ferrous metals. This is a low-cost and fast procedure that develops corrosion and wear resistance on the surface [1,2,3,4,5] while also improving the adhesive and lubricating properties of the material. For that reason, phosphating plays a very important role in the automobile, processing and domestic appliances industries [6] to protect steel [7] and its alloys against corrosion. The use of phosphating on steel has been shown in Table 1.

Other than that, phosphate coatings are also prepared to cover alloys of such metals as zinc, cadmium, aluminum and magnesium. Phosphating creates a protective layer on the surface once the metal matrix is immersed in a phosphate solution as a result of chemical reactions between a dilute solution of phosphoric acid and, for example, zinc, iron and manganese. The phosphating mechanism is described by Yan et al. in paper [8]:

(1) $$Fe\to {Fe}^{2+}+2e^{-}$$

(2) $$H^{+}+2e^{-}\to H_{2}↑$$

(3) $${HPO}_{4}^{2-}+{Me}^{2+}+yH_{2}O\to MeHPO_{4}\cdot yH_{2}O$$

(4) $$2PO_{4}^{3-}+{xMe}^{2+}+yH_{2}O\to Me_{x}(PO_{4}{)}_{2}\cdot yH_{2}O$$

where Me^2+^ represents metal cations Zn^2+^, Fe^2+^, Mn^2+^ and Ni^2+^.

The composition of the phosphate bath affects the properties of the resulting coatings. According to the authors of [9], the addition of sodium molybdate to the bath increased corrosion resistance of the coatings. They found that the corrosion current decreased with increased content of Na_2_MoO_4_. In this case, the coating was used as an intermediate protective layer to improve the adhesion of the final paint layer to automobile iron castings. The researchers conducted two tests using (a) salt spray and (b) atmospheric species. The anti-corrosion effect on car castings was demonstrated in both cases. Unalloyed steels are more often subjected to the phosphating process than alloyed ones, because the presence of a passive oxide layer on the surface of corrosion-resistant steels makes them less susceptible to phosphating. However, some research on phosphating alloyed steels has been conducted [10]. Oskuie et al. [10] reported that tri-cation phosphate coating of Zn, Ca and Fe was grown electrochemically on 316 steel. A cathodic current was used as an accelerator for the phosphating process. The higher electrophosphating current density was shown to cause finer coating crystals that deteriorated the quality of the layer. Manna [11] tested phosphate coatings on steel with a ferritic–pearlitic structure, tempered martensite and tempered martensite with an oxide layer. In order to form the coating, a bath free from nitric acid was used. The test results showed that the structure of the substrate affected the thickness of the deposited phosphate coating. In turn, Ivanova [12] tested phosphate coatings based on pure Zn and Zn + Mn mixtures grown on carbon steels to determine the thickness of the coatings and the extent to which the core of the material (substrate) dissolved. It turned out that manganese phosphate greatly affected the obtained coating, reducing its thickness regardless of solution concentration and temperature that ended up in an increase of the mass of dissolved substrate metal. The coatings deposited in Zn-Mn baths consisted of the following phases: hopeite, phosphophyllite, quasihopeite, strunzite and their mixtures. Borko et al. [13] described how Domex 700 steel behaved in a 0.1 M NaCl environment. Prior to corrosion resistance measurements, the surface of the steel was sequentially treated by means of (1) grinding, (2) phosphating and (3) shot-peening. It turned out that the obtained MnP coating evenly and continuously covered the entire substrate both after grinding and shot-blasting. However, a more uniform layer with fewer defects was obtained after grinding. In turn, the shot-blasting contributed to the deterioration of thermodynamic and kinetic stability (corrosion resistance) of the coatings, with the opposite effect achieved after grinding and manganese phosphating. Taking into account the morphology and weight of phosphate coatings, three stages of the process can be distinguished: (1) corrosion of the substrate, (2) nucleation of the isolated phosphate crystals and (3) growth of the continuous phosphate coating [14]. Deposited coating is composed of crystals of disubstituted and trisubstituted metal phosphates [7]. On the other hand, Fang et al. [14] demonstrated that the deposited coating contained many close-packed lump crystallites mainly composed of (Mn,Fe)_5_H_2_(PO_4_)_4_·4H_2_O complexes.

Fractal analysis is becoming increasingly popular in research of the outer surface layers [15], because it gives insight into various aspects of the geometric structure extending over several orders of magnitude. It is also possible to learn the relationships between the fractal and stereometric characteristics of the technological surface layers. In that framework, each surface can be characterized in terms of a single parameter—fractal dimension. Estimation of fractal dimensions is relatively simple and enables the analysis of surface variability from various images obtained, among others, using SEM microscopy [16]. Rovani et al. in paper [17] published results on phosphating of AISI steels previously subjected to heat treatment: hardening + tempering followed by shot-blasting. In the next steps, several different layers were applied: a zinc phosphate, a phenolic resin (base varnish) and a topcoat based on MoS_2_. It was shown that phosphating itself significantly influenced the surface texture of the resin-bonded coating, taking into account changes in the surface texture ratio (S_tr_ parameter). Kurella et. al. in paper [18] showed that fractal dimensional analysis helps to interpret multi-scale surfaces. Moreover, fractal dimension analysis allows for the study of the surface of the materials in terms of topographic and chemical changes [19].

The aim of this paper was application of fractal analysis to SEM images in order to characterize the geometric structure of the phosphate layer, that is, to show that the quality of the obtained coatings can be determined through fractal analysis To this end, two steel samples that differ significantly in their chemical composition were selected and their spatial structures of the surface layers were determined. Such characterization might be also useful to optimize manufacturing processes or to identify the degradation effects.

## 2. Materials and Methods

Two steel samples were investigated in this study: X39Cr13 (corrosion-resistant martensitic steel [20]) and S355J2 (non-alloyed structural steel [21]). The chemical composition of both materials is presented in Table 2.

**Figure 1:**
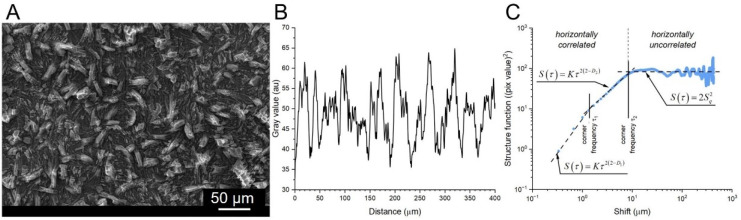
(**A**) SEM image of S335J2 steel under ×300 magnification and its row-averaged mean roughness profile (inset) SEI 20 kV, WD 10 mm, SS 44; (**B**) row–averaged original SEM image showing mean roughness profiles; (**C**) log–log plot of the structure function vs. horizontal shift obtained from the roughness profile (S is the RMS of pixel intensity). The corner frequency separates horizontally correlated surface bumps from the uncorrelated ones.

In the beginning, both steel samples were heat-treated in accordance with the specific processing guidelines. On one hand, the sample of X39Cr13 steel was hardened for 20 min at the austenitization temperature of 1050 °C followed by tempering for two hours at 300 °C. On the other hand, the sample of S355J2 steel was annealed for two hours at a temperature of 890 °C. Then, the surfaces were cleaned to remove residues and contaminants and degreased to allow the phosphate coating to be deposited. Prior to phosphating, prepared surfaces were activated in an aqueous solution (10%) of hydrochloric and sulfuric acids. Activation took 5 min at room temperature (22 °C). Finally, the phosphating was performed in a bath containing MnHPO_4_ (3 g/L), Mn(NO_3_)_2_ (10 g/L), ZnO (5 g/L), H_3_PO_4_ (20 g/L), NaF (1 g/L). The process parameters were as follows: T = 52 °C, t = 1 h. The tests were performed both on the processed surfaces and in the cross-sections of the samples. For this purpose, square pieces measuring 10 × 10 mm^2^ were cut out from the samples. The preparation of the metallographic specimens involved grinding and polishing. Selected samples were also etched in 5% nitric acid (for non-alloyed steel) and iron chloride (for alloyed steel). Detailed studies included observations in light microscope (LM Olympus GX41) and scanning electron microscope (SEM Jeol JSM-6610L) for metallographic examinations, chemical composition analysis and fractal analysis. The XRD experiments were carried out on a Seiffert 3003T/T diffractometer. A CoKα radiation cobalt lamp was used (λ = 1.79026 Å). The X-ray tube was operated at 40 kV and 30 mA. The XRD patterns were collected in 2 ranges between 5° and 90°. SEM images are composed of grayscale pixels corresponding to pseudo-heights that can be processed in order to derive various characteristics of the surface geometry. In the present paper, the two following approaches were used: (1) fractal analysis that makes use of scaling invariance between samples of averaged surface profiles [24,25,26,27,28,29,30] and (2) statistical approach working on the separated segments of original images. In the first method, original SEM images (example shown in Figure 1A) were averaged along the rows of the slow scan axis in order to obtain roughness profiles (Figure 1B), which were then processed into discrete structure functions according to the formula [31]:

(5) $$S\left(\tau \right)=\frac{1}{N-m}\sum_{n=1}^{N-m}{\left(z_{n+m}-z_{n}\right)}^{2}$$

where *τ* is the discrete shift between original profile and its copy, *m* = *τ*/Δ is the integer number, Δ—the scan step, *z*_k_—the k-th sample of the mean profile and *N*—the number of samples in each profile. Figure 1C shows the plot of the structure function for S355J2 steel under 300× magnification. Thomas and Thomas [32] showed that for sufficiently small shifts *τ*, the one-dimensional structure function obeys the power–law dependence in the form:

(6) $$S\left(\tau \right)=K\tau^{2(2-D)}$$

where *D* is the unitless quantity referred to as fractal dimension, and *K* is the scaling factor referred to as pseudo-topothesy. Any sharp change in the slope of the log–log plot of the structure function vs. shift establish the corner frequency *τ*_c_, which separates segments of different scale-invariance characteristics.

In the second method, surface morphology was analyzed by means of statistical shape analysis. To this end, SEM images were segmented using the watershed algorithm followed by determination of shape descriptors for planar figures: circularity, roundness, solidity, Feret’s diameter and watershed diameter. Circularity is a positive fractional number that exhibits the deviation from a perfect circle. It is calculated according to the formula:

(7) $$C=\frac{4\pi A}{P^{2}}$$

where *A*—is the segment area and *P*—its perimeter. When the circularity decays to zero, the figure becomes increasingly elongated, and when it comes close to unity, a perfect circle appears. A similar measure is a roundness that equals the ratio of the lengths of the minor and the major semi-axes of the best fit ellipse replacing given selection area:

(8) $$R=\frac{a_{min}}{a_{max}}=\frac{A}{\pi a_{max}^{2}}$$

where *a*_min_, *a*_max_ are the minor and major semi-axes of the equivalent ellipse, respectively. In turn, solidity is the ratio of the actual area of the figure and its convex hull:

(9) $$S=\frac{A}{A_{CH}}$$

For a perfectly convex figure, solidity equals one; otherwise it is less but non-zero. The last two parameters define specific size of the segments in terms of various lengths. On one hand, the watershed diameter is the diameter of the equivalent circle (same area as a given figure):

(10) $$d_{WS}=\sqrt{\frac{4A}{\pi }}$$

On the other hand, Feret’s diameter *d*_F_ equals the distance connecting any two points on the boundary of a segment. Among all possible *d*_F_ values, the minimum and maximum Feret’s diameters are of special importance for characterization of particle shape and form.

## 3. Results and Discussion

**Figure 2:**
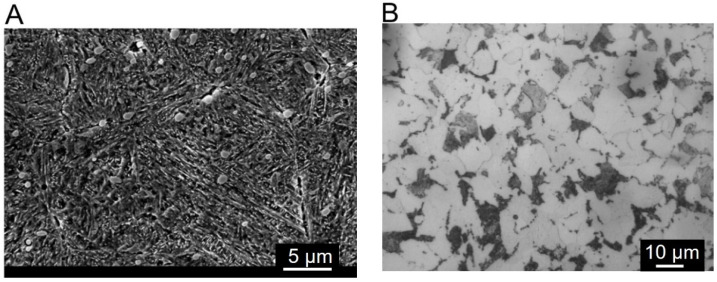
Structure of steel: (**A**) SEM image of X39Cr13, (**B**) optical microscope image of S355J2.

The structures of the steel samples after heat treatment are shown in Figure 2. The structure of the X39Cr13 steel was tempered martensite with carbide precipitates (Figure 2A), while that of S355J2 was ferrite–pearlite (Figure 2B).

**Figure 3:**
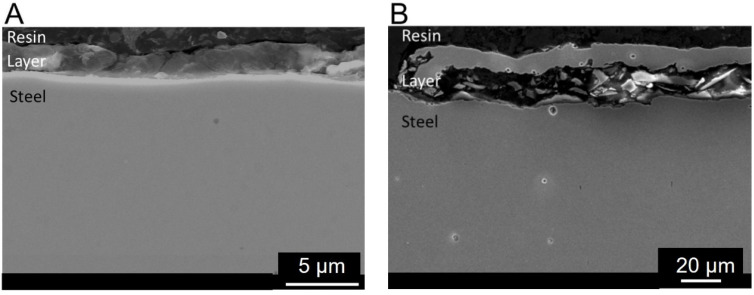
SEM images of phosphate layers deposited on steel samples: (**A**) X39Cr13, (**B**) S355J2.

Recorded images were then used to determine the structure of the deposited phosphate coatings (Figure 3). The thickness of the coating on martensitic steel was one order smaller (~3 μm) compared to that on non-alloyed steel (~30 μm).

**Figure 4:**
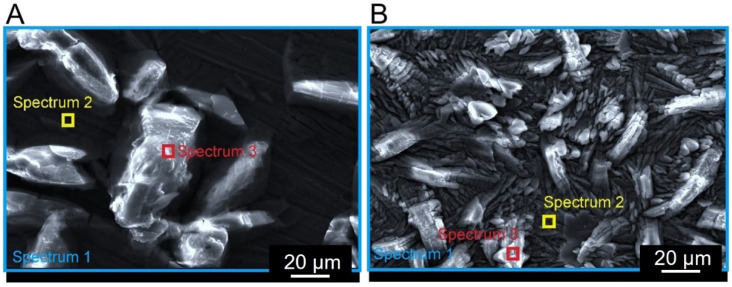
Steel surface after phosphating with a point and area spectrum markings: (**A**) X39Cr13 steel, SEM (SEI 30 kV, WD 10 mm, SS 46 (**B**) S355J2 steel, SEM (SEI 20 kV, WD 10 mm, SS 44).

SEM images exhibit the crystalline structure of phosphate coatings that were made of phosphate crystals in the form of needles. A similar structure of the phosphate layer was observed by Rovani et al. [17]. Another paper reported that coatings based on zinc and phosphorus showed a predominance of needle-like crystals [33]. Some researchers identified the structure of this coating seen in SEM images as scale-like crystal structure [34]. Rossi et al. [35] demonstrated that phosphate-converted samples exhibited highly irregular surface structures, which turned out typical for this type of coating. Additionally, the thickness of the layer appeared to be between 5 and 10 μm [35]. Phosphating in an environment containing zinc and manganese ions resulted in a coating composed mainly of metal phosphates. Microscopic observations of phosphated steel surfaces showed differences in the morphology of these layers. The coating produced on X39Cr13 steel (Figure 4A) was characterized by much lower density than the coating produced on S355J2 steel (Figure 4B).

Phosphating of non-alloyed steel resulted in the formation of a much more compact deposit compared to that on alloyed steel. The results of the chemical analysis of the surfaces of tested coatings (Table 3) confirmed the microscopic observations.

**Figure 5:**
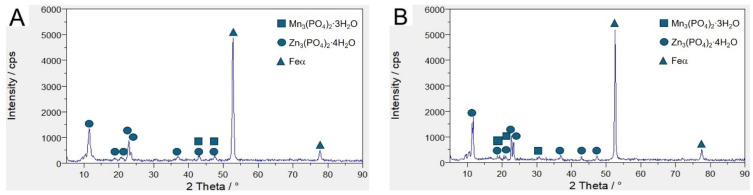
X-ray diffractogram: (**A**) X39Cr13 steel; (**B**) S355J2 steel.

Significant differences in chemical composition of the deposited coatings were observed in X39Cr13 steel. EDS spectra taken from the needle (Figure 4A, Spectrum 3) appeared significantly different from those in the neighboring area (Figure 4A, Spectrum 2). The needles show significantly lower amounts of such elements as oxygen, phosphorus, manganese and zinc. On the other hand, an abundance of elements constituting the steel itself, such as iron and chromium, was observed in these parts of the samples. This shows that the coating made on alloyed steel is less tight than that on non-alloyed steel. The obtained XRD (Figure 5) results showed that the phosphate layer was composed of Zn_3_(PO_4_)_2_·4H_2_O and Mn_3_(PO_4_)_2_·3H_2_O. The zinc-based compound predominated to a large extent. Using a phosphorus bath with a similar chemical composition, Nguyen et al. [36] showed that in coatings with a ZnO content greater than 3 g/L contains mixed phases. There are, among others, compounds such as Mn_3_(PO_4_)_2_·3H_2_O and Zn_3_(PO_4_)_2_·4H_2_O. The XRD diagram presented by the researchers shows that there is only one reflection from which the Mn(PO_4_)_2_·3H_2_O phase originates. The remaining picks come from a zinc-based compound.

**Figure 6:**
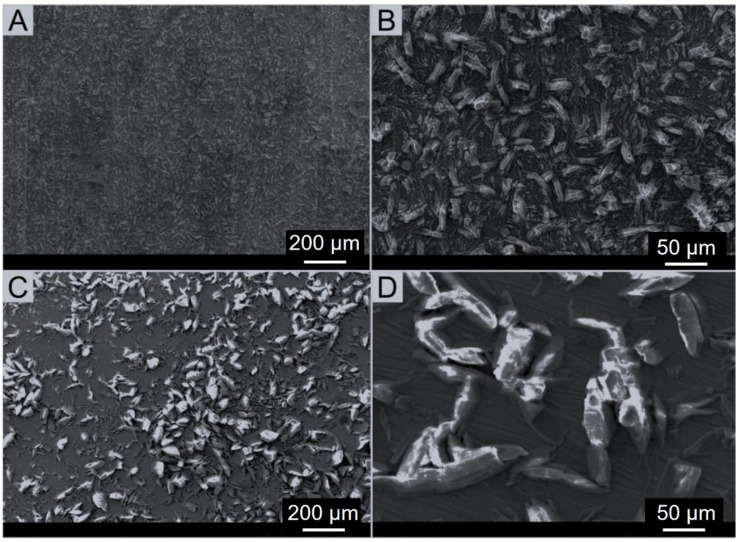
SEM images of steel samples under various magnifications: (**A**) S355J2 ×75 magnification, SEM (SEI 20 kV, WD 10 mm, SS 44), (**B**) S335J2 ×300 magnification, SEM (SEI 20 kV, WD 10 mm, SS 44), (**C**) X39Cr13 ×75 magnification, SEM (SEI 30 kV, WD 9 mm, SS 46), (**D**) X39Cr13 ×300 magnification, SEM (SEI 30 kV, WD 9 mm, SS 46).

The thinner phosphate layer on martensitic steel is probably due to its previous passivation, which in general has a beneficial effect [37]. In this case, however, passivation adversely affects the applied top layer. Due to the presence of a passive layer, the phosphating process on X39Cr13 steel was worse. According to the literature [38], the phosphating process involves dissolving a given metal in an acidic solution of soluble primary phosphates. The next process is the hydrolysis of these phosphates, which leads to the precipitation of insoluble tertiary phosphates. For the phosphating process to proceed properly, the metal should dissolve at a moderate rate, which will enable the necessary neutralization and supersaturation of the near-surface solution. Therefore, the phosphating process is deteriorated due to the presence of elements such as nickel, chromium or molybdenum in the composition of the steel. A smaller amount of precipitated phosphate is then produced. The literature states [10] that phosphating would significantly improve by the break of the chromium oxide layer. In contrast, no such large differences in the chemical composition of the coating on S355J2 steel were noted (Table 3, Figure 4B, Spectrum 2 and 3). EDS analysis also exhibits discontinuous coating layer that agrees with previous studies [17]. One possible explanation is the substrate cleaning process, which affects the nucleation and hence formation of zinc phosphate on the surface. In addition, in different morphologies of phosphate coatings applied to non-alloyed steel, substrates may be the result of the use of different phosphating baths. This also affects the different porosity of these coatings. According to researchers [33], the lowest porosity was in the coating made from a solution of zinc phosphate with ammonium niobium oxalate and benzotriazole. Significantly greater porosity was found in the zinc phosphate coating. In turn, the addition of niobium to the phosphating baths reduced the porosity of the coatings. Figure 6 presents grayscale SEM images of the investigated steel specimens: S355J2 (non-alloy quality structural steel) and X39Cr13 (martensitic stainless steel) viewed at two magnifications: ×75 and ×300.

Visual comparison of these images reveals the following similarities: both samples have a coarse surface covered with sharp precipitates, which are otherwise randomly oriented. Apart from that, however, the size, the shape and the alignment of these precipitates appear notably different. As a matter of fact, in the S355J2 sample shown in Figure 6A,B, the predominant geometrical forms are oblong polygons distributed evenly over the surface, the size of which ranges from a fraction of a micrometer up to few tens of micrometers, and the aspect ratio (the ratio of the shortest and the longest Feret’s diameters) is between 1:3 and 1:5. Unlike that, on the surface of X39Cr13 steel sample in Figure 6C,D such precipitates can be seen that take on much more diverse and irregular shapes and might be even one order of magnitude bigger than those in the previous specimen. In addition, they are not distributed homogeneously over the surface, but instead they appear to agglomerate, forming clusters few hundreds micrometers in diameter.

Table 4 presents fractal parameters describing surface height variations in terms of allometric scaling that were derived from SEM images recorded at two magnification levels.

In the case of the S355J2 steel sample, multifractal behavior can be seen related to alignment patterns at different scale lengths. The lower scaling range, limited by the corner frequency τ_1_ and defining the size of the lowest geometrical forms on the surface, extends up to 1.41 and 8.14 μm, viewed at ×300 and ×75 magnifications, respectively. On the other hand, the upper scaling ranges established by the corner frequencies τ_2_ approach 8.34 and 587 μm, analyzed at ×300 and ×75 magnifications, respectively. Note, however, that when the resolutions are taken into consideration, then the frequencies τ_1_ in both images correspond to ca. 5 pixels in each image regardless of the magnification, which might be a fingerprint of inevitable signal noise or high-frequency surface roughness. Note also that the scaling behaviors of the image data at both magnification levels overlap, which means that the corner frequency τ_1_ in the low resolution image (×75) equals that of τ_2_ in the high resolution image (×300). The same observation can be seen when comparing the values of fractal dimensions, because the fractal dimension D_1_ = 2.41 in the low resolution image (×75) is nearly equal to D_2_ = 2.42 in the high resolution image (×300). Such a finding might lead to a conclusion on the average size of the basic bumps on the surface of S355J2 steel, which take on elongated figures ca. 1 μm wide and ca. 8 μm long. At lower magnification, however, these bumps are found to agglomerate into clusters almost two orders of magnitude wider (600 μm in horizontal diameter). The fractal dimension was also determined by Paun et al. [24] and Kong et al. [25] using SEM images. Results for X39Cr13 steel presented in Table 3 exhibit significantly different scaling behavior, in which both corner frequencies and fractal dimensions appear similar regardless of the magnification: the corner frequency τ_1_ explaining small bumps approaches ca. 6 μm, while the corner frequency τ_2_ corresponding to the larger bumps is ca. 30 μm. As a result, fractal analysis reveals the appearance of oval bumps, which are around 4 times larger in their linear dimensions compared to those of the previous sample. Farias et al. [34] showed that the zinc phosphate coating had a roughness R_a_ of 0.47 μm and the crystals were 24.3 μm in diameter.

To verify the results of the fractal approach, additional analysis was carried out relying on the separation of SEM images into a series of touching segments followed by statistical analysis of their form and size to determine such shape descriptors as circularity, roundness and solidity, together with specific size parameters such as Feret’s diameter and watershed equivalent diameter. According to the literature [26], the Feret diameter of a particle can be defined as the distance between two parallel tangent boundaries of the object. In particular, the maximum and minimum Feret diameters, are often used for the characterization of particle sizes [27]. According to the literature [28], the minimum and maximum Feret diameters of objects are compared according to the aspect ratio, whereas the axial ratio refers to the best match between the minor and major axes of the ellipse. Detailed results are summarized in Table 5, where appropriate mean values are presented, and in Figure 6, where all data points are shown in a graphical form to reveal appropriate statistical distributions of the quantities under study together with their mean values and corresponding standard deviations. Note that SEM images with different magnification were chosen for this analysis (×300/S355J2 vs. ×75/X39Cr13) to ensure similar number of segments established in the scan area.

Comparison of obtained means proves that the predominant shape of the average segments does not vary between specimens under study. As a matter of fact, circularity equal to ca. 0.7 and roundness equal to ca. 0.6 both imply the elongated shape of such a figure, while solidity larger than 0.8 clearly points at its convex habit. Together, all three descriptors strongly suggest a nearly oval shape of the average segment. The only difference lies in the size of the segments, which agrees well with the previous findings from the fractal analysis. As a rule, both the Feret’s diameter and the watershed diameter demonstrate that the size of specific bumps on the surface of S355J2 steel is roughly one order of magnitude lower than those in X39Cr13 (1.4/2.1 μm vs. 20/12 μm, respectively). 

**Figure 7:**
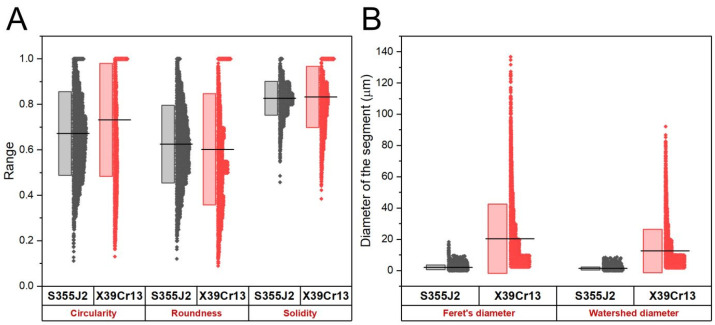
(**A**) Half-box plots of shape descriptors of the segments outlined in SEM images by means of the watershed algorithm: circularity (equation number 34), roundness (equation number 7) and solidity (equation number 8) and (**B**) comparison of the distributions of specific size descriptors: Feret’s diameter and watershed diameter (equation number 9). Closed dots show data points and straight lines correspond to mean values, while the heights of the boxes extend to ± standard deviation.

In order to verify how the results for particular segments are distributed, Figure 7 shows half-box plots of the image data. Presented graphs confirm previous findings as to similarity of the shape descriptors: circularity, roundness and solidity and notable difference in the size of the established segments. Circularity is an important geometrical parameter in evaluation of grain shapes, as it might provide an insight into object roundness [28]. As indicated in the literature [29], surface roughness is combined with irregularity related to the level of roundness of natural grains. The plot of estimated diameters of the segments on the surface of S355J2 steel specimen exhibits quite uniform although narrow distribution of the data points with sharp edges on both sides, which might be concluded in high identity of the sizes of the segments. In contrast, the distributions of the same data on the surface of X39Cr13 steel appears strongly asymmetric, with a flat edge at the bottom, but a very long tail extending up to 100 μm at the top. This notable asymmetry is due to a large variation in the diameters of the segments, established in the image without any significant change in their habits according to the results of the shape descriptors. This allows us to refer to this surface as self-affine (self-similar within a limited range of scale lengths).

## 4. Conclusions

Phosphate coatings on alloyed and unalloyed steel samples exhibit crystalline structure. This coating was composed of Zn_3_(PO_4_)_2_·4H_2_O and Mn_3_(PO_4_)_2_·3H_2_O, with a significant advantage of the first phase. Much better quality of the phosphate coating was obtained on S355J2 steel. Comparative analysis by means of fractal parameters and shape descriptors reveal morphological differences between specific geometrical features of predominant forms on the surface of S355J2 and X39Cr13 steel samples. Fractal analysis uncovers aggregated structure of the surface bumps in S355J2 steel, but self-affine in X39Cr13 sample; fractal parameters reflect the appearance of oval bumps, which are around 4 times larger in their linear dimensions on the surface of S355J2 compared to X39Cr13. Statistical analysis of the shapes and sizes of the segments established in SEM images using the watershed discrimination algorithm generally confirms the findings of the fractal approach: the average segment takes on an oval shape regardless of the sample; however, its size on the surface of S355J2 steel is roughly one order of magnitude lower than that of X39Cr13. As a matter of fact, the surface of S355J2 might be referred to as clustered, while that of X39Cr13 can be regarded as self-affine. Microstructural tests combined with EDS analysis allowed for the conclusion that the phosphate coating is more tight and compact in the case of S355J2 steel. In this case, the formed phosphate layer is characterized by a strong bond with the substrate. Therefore, it can be used as a base, e.g., for a paint coating. Geometric characteristics of the structure may be desirable both to optimize production processes and to understand the effects of material deterioration.

## Figures and Tables

**Table 1:** Application of phosphate coatings for steel.

Application of Phosphate Coatings	
Steel	A layer facilitating cold-forming of steel. The phosphate coating in this case acts as a layer that prevents contact between the processed steel and the material from which the tool is made.
	Temporary protection of products during transport, storage and operation
	Anti-friction layer—reduces the coefficient of friction and also reduces the wear of interacting parts. This coating prevents welding of mating metals and quiets the element’s operation system. Moreover, it reduces surface irregularities after mechanical processing and shortens the running-in period.
	Primer layer—increases the corrosion properties and adhesion of paint coatings. Corrosion resistance increases significantly after covering it with a layer of oil, paint or varnish.
	Insulating layer.

**Table 2:** Chemical composition of the steels under investigation.

Type of Steel	Acc. to	Chemical Composition, % Mass.					
		C	Si	Mn	P	S	Cr
X39Cr13	analysis	0.42	0.39	0.55	0.020	0.004	13.73
	EN 10088-2 [22]	0.36 ÷ 0.42	max. 1.00	max. 1.00	max. 0.040	max. 0.015	12.50 ÷ 14.50
S355J2	analysis	0.16	0.42	1.43	0.021	0.024	-
	EN 10025-2 [23]	max. 0.20	max. 0.55	max. 1.60	max. 0.025	max. 0.025	-

**Table 3:** Analysis of the chemical composition (EDS) of the surface of the phosphate coating deposited on X39Cr13 and S355J2 steel samples.

Element	Weight, %					
	Spectrum 1		Spectrum 2		Spectrum 3	
	X39Cr13	S355J2	X39Cr13	S355J2	X39Cr13	S355J2
O	22.86	32.87	5.06	31.40	39.76	37.11
P	9.56	15.46	1.17	12.45	17.35	16.72
Si	-	-	0.35	-	-	-
Cr	6.43	-	13.66	-	-	-
Mn	2.75	4.24	0.59	2.10	3.58	3.33
Fe	33.91	10.09	77.90	30.10	1.73	8.80
Ni	0.83	-	-	0.36	0.70	0.42
Zn	23.66	36.90	1.27	23.02	36.88	33.19
Ca	-	0.45	-	-	-	0.24
Ti	-	-	-	-	-	0.19
Cu	-	-	-	0.58	-	-

**Table 4:** Results of fractal analysis of SEM images of steel samples under investigation: D_1_, D_2_—fractal dimensions, τ_1_, τ_2_—corner frequencies.

Sample	Magnification	Image Resolution [μm/px]	D_1_ [-]	D_2_ [-]	τ_1_ [μm]	τ_2_ [μm]
S355J2	×75	4/3	2.49	2.83	8.14	587
	×300	1/3	2.19	2.42	1.41	8.34
X39Cr13	×75	4/3	2.16	2.45	7.57	32.4
	×300	1/3	2.11	2.35	5.26	29.4

**Table 5:** Mean shape descriptors of the segments outlined in SEM images using the watershed algorithm.

	S355J2 (×300)	X39Cr13 (×75)
Circularity	0.67 ± 0.18	0.73 ± 0.25
Roundness	0.63 ± 0.17	0.60 ± 0.25
Solidity	0.83 ± 0.07	0.83 ± 0.13
Feret’s diameter [μm]	2.1 ± 1.6	20 ± 22
Watershed diameter [μm]	1.4 ± 1.0	12 ± 14

## Data Availability

The data presented in this study are available on request from the authors.
